# Pemphigus vulgaris aggravated by obsessive-compulsive behavior: the importance of adjuvant topical occlusive dressing^☆^^☆☆^

**DOI:** 10.1016/j.abd.2020.06.026

**Published:** 2021-05-15

**Authors:** Paula Basso Lima, Marilia Formentini Scotton Jorge, Luciana Patrícia Fernandes Abbade, Sílvio Alencar Marques

**Affiliations:** Faculty of Medicine, Universidade Estadual Paulista, Botucatu, SP, Brazil

Dear Editor,

Pemphigus vulgaris (PV) can be a difficult clinical diagnosis if mucosal involvement is not present. The occurrence of IgG4 anti-Dsg1 autoantibodies is associated with the pathogenesis of skin lesions and anti-Dsg3 with mucosal lesions. Serologically, the predominantly cutaneous presentation has circulating anti-Dsg1 and anti-Dsg3 autoantibodies, with a tendency to higher titers of anti-Dsg1 than anti-Dsg3, which implies a rare clinical phenotype of pemphigus vulgaris.^1^

This is a case report of a 64-year-old male patient with a history of depression, type 2 diabetes mellitus, alcoholism, and liver cirrhosis. He was referred, with a previous diagnosis of PV, due to difficulties in therapeutic management and with a suggestion for rituximab therapy. He had numerous ulcerated lesions, covered by hemato-meliceric crusts, predominantly on the face, pinna and cervical region (Fig. 1). No mucosal lesions were observed. Due to the exuberance of the condition with an atypical clinical presentation, new biopsies were performed, which confirmed the diagnosis of PV through histopathology and direct immunofluorescence. The clinical and laboratory investigation corroborated the aforementioned comorbidities. Serologies for hepatitis and HIV infection were negative. The patient had been using prednisone 0.85 mg/kg for two years without improvement.

**Figure 1:**
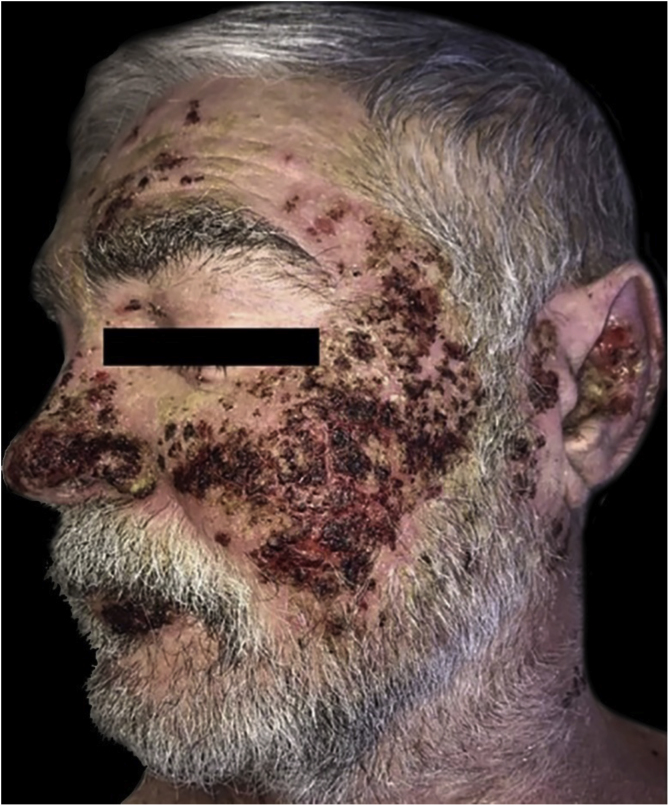
Pemphigus vulgaris and obsessive-compulsive behavior. The multiple lesions are covered by hemato-meliceric crusts on the face.

During the hospitalization, a compulsive, daytime pattern of lesion manipulation was identified, which resulted in the exacerbation of the pre-existing lesions and the formation of crusts on them, which apparently justified the lack of response to treatment. After a psychiatric evaluation, sertraline 50 mg/day was started, together with psychotherapy, and dressing in polyhexamethylene biguanide (PHMB) gel, rayon and occlusion (Fig. 2). It was also decided to add azathioprine 150 mg/day and maintain the prednisone dose. There was an immediate and visible improvement after two days of the established therapy, and a significant improvement after 40 days (Fig. 3).

**Figure 2:**
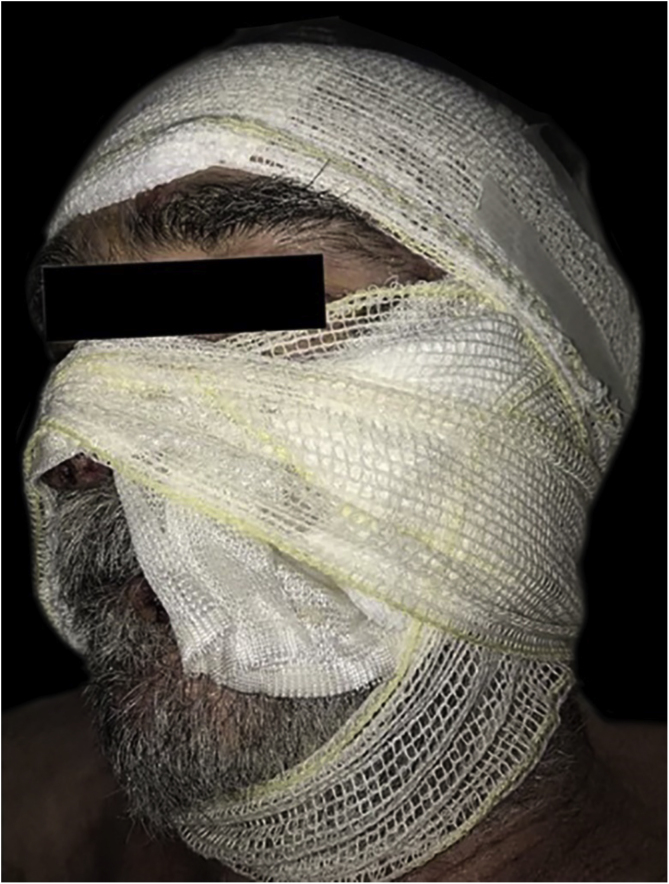
Pemphigus vulgaris and obsessive-compulsive behavior. Dressing with PHMB gel, rayon and occlusion dressing.

**Figure 3:**
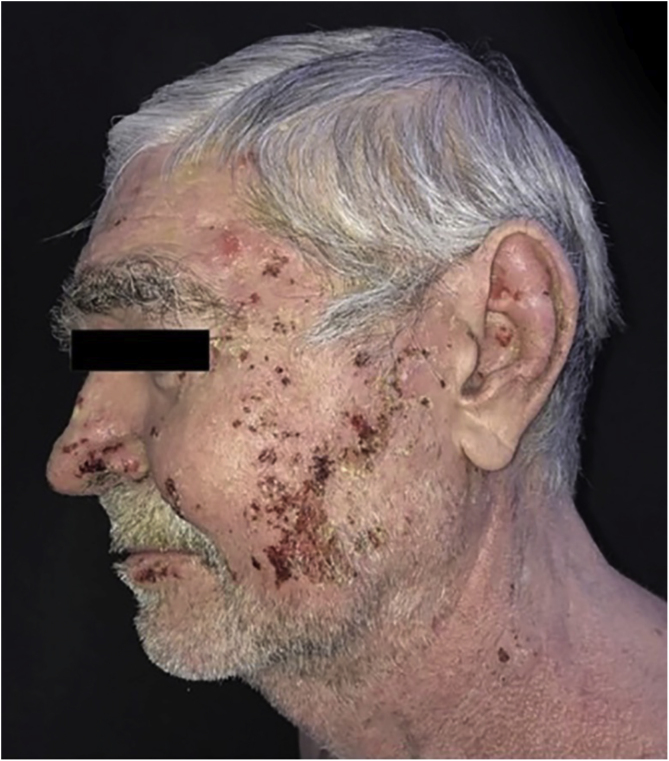
Pemphigus vulgaris and obsessive-compulsive behavior. Evolution after 40 days of the established therapy.

A follow-up study of patients with pemphigus (broad sense) found an incidence of depression 1.98 times more frequent than in the control group and 2.42 times higher when pemphigus was associated with low income. This higher frequency of depression would be associated with the chronic, relapsing, stigmatizing and debilitating course of the disease.^2^ compulsive disorders (OCD), in this case, compulsive and repetitive excoriation impulse control disorder, often begins after a previous dermatological condition, with the face being the area most preferably involved.^3^ Associated with the diagnosis of OCD, a high frequency of anxiety disorders was observed in 79.6% of cases and substance abuse disorders in 38.6% of patients.^4^

In the reported case, psychotherapeutic and pharmacological support, local care and use of an occlusive dressing that prevented local manipulation were essential for the success of the treatment. The dressing was changed daily, with topical care, use of PHMB and under medical and nursing supervision.

Corticosteroid therapy is the first choice for pemphigus, with the frequent help of adjunctive corticosteroid-sparing therapies.^5^ Recently, rituximab has been suggested as a first-line drug for severe or recalcitrant cases.^5^ The present report aimed to highlight the importance of identifying psychological disorders associated with dermatological diseases, appreciate the global care of the patient and call attention to the importance of complementary topical care, little valued in clinical practice.

## Financial support

None declared.

## Authors’ contributions

Paula Basso Lima: Approval of the final version of the manuscript; design and planning of the study; drafting and editing of the manuscript; intellectual participation in the propaedeutic and/or therapeutic conduct of the studied case; critical review of the literature.

Marilia Formentini Scotton Jorge: Approval of the final version of the manuscript, critical review of the manuscript.

Luciana Patrícia Fernandes Abbade: Approval of the final version of the manuscript; intellectual participation in the propaedeutic and/or therapeutic conduct of the studied case; critical review of the manuscript.

Sílvio Alencar Marques: Approval of the final version of the manuscript; drafting and editing of the manuscript; critical review of the literature, critical review of the manuscript.

## Conflicts of interest

None declared.
